# Gaining reconciliation when living with insulin treated diabetes: a qualitative study using content analysis

**DOI:** 10.1080/17482631.2022.2090659

**Published:** 2022-06-20

**Authors:** Susanne Eriksson, Lena-Karin Gustafsson

**Affiliations:** School of Health, Care and Social Welfare, Mälardalen University, Eskilstuna, Sweden

**Keywords:** Acceptance, disease management, diabetes mellitus, experiences, living circumstances changes, qualitative content analysis

## Abstract

**Purpose:**

The aim was to describe experiences of the reconciliation process when living with insulin treated diabetes.

**Methods:**

The study has a qualitative descriptive design, based upon nineteen in- depth interviews with persons diagnosed with insulin treated diabetes, analysed using qualitative content analysis.

**Results:**

The study show the reconciliation process during different time periods that appeared as domains in the interviews. The time at diagnosis showed experiences of striving for control getting insights and knowledge. It meant striving for control of life circumstance changes, supported by professionals but also from others. In Presence showed developing strategies as a tool struggling for balance in body and life and the need of evaluating relations to others. Future was sometimes avoided as this might lead to speculations about a future life with threats and uncertainty about disease complications, as well as adaption. This meant on the same time an uncertainty, as a degree of risk-taking and hope for the best.

**Conclusions:**

Persons with insulin treated diabetes need to develop flexible strategies for daily life to continuously re-evaluate their planning for attaining reconciliation. A conclusion is also that these persons need to develop a flexible regime that facilitates both quality of life and medical outcomes to reach reconciliation.

## Introduction

Diabetes mellitus is a common disease, and without treatment leads to severe and life-threatening multiorgan sequela Leslie et al., 2016. The complexity of the therapeutic regime in diabetes, together with a lack of adequate knowledge, makes it very difficult for some people to adhere to the restrictions of living with insulin treated diabetes. Some people find the introduction of insulin a major crisis, with the resulting loss of control over their body Vermeire et al., 2003.

Health-care professionals play an important role in decision-making when patients consult them with questions related to their diabetes. Health-care professionals are supposed to help them develop preventative strategies to be able to avoid, for example, a hypoglycaemic event Tan P et al., 2012.

The person with diabetes has no other choice than to adapt to their illness. Denial of the disease can lead to false reconciliation Johansson et al., 2009. When the diagnosis comes, some persons are prepared, others are not. Some have experience of diabetes from their family and adapt to living circumstances changes overnight. Others don’t experience that they have diabetes, even though they received their diagnosis which does not motivate lifestyle changes; Smide & Å, 2009. For some, the diagnosis comes as a shock, others experience relief about getting an explanation of the imbalance in the body that they have experienced for some time Johansson et al., 2009. Listening to signs from the body might later become a tool for decision-making when living with diabetes Kneck et al., 2012. Timely and tailored information and support over time can help persons with inulin treated diabetes develop a flexible regime to handle both quality of life and medical treatment; Burridge et al., 2016; Frost et al., 2014. Support over an extended period from both health-care professionals and relatives is important for the patient’s self-management ability and diabetes empowerment; Isaksson et al., 2015; Huston et al., 2016; Audulv et al., 2012. In order to not become your own illness, it is important that support is not experienced as a strain but is based on individual support needs and openness; Johansson et al., 2009; Burridge et al., 2016; 2016.

Effective partnerships with health-care professionals are important, for young persons with diabetes. They don’t want authoritarian support in their interactions with healthcare professionals, but they do need emotional support and a space for development on their own terms; Karlsson et al., 2008. They also need to meet others of the same age to successfully manage the illness Scholes et al., 2012 and increase the ability to self-manage the disease; Audulv et al., 2012.

The duration of the illness is not always important for its understanding as even years after diagnoses, some can find it hard to find balance in life Kneck et al., 2012. Living with diabetes means taking responsibility for balancing plasma glucose and incorporating the illness into a life which necessitates gaining knowledge and sensitivity to changes; Mishalia et al., 2011, as well as the fear of losing control, and future complications; Mishali et al., 2011. Those who have formed positive images of their illness tend to view it as something they must live with, and they seem to accept the condition as a part of themselves and new way of being. Earlier research showed that denial of the disease could lead to acceptance of the disease but not a real reconciliation with the life that lives; Kneck et al., 2012; Burridge et al., 2016. Those who form negative images tend to keep the disease hidden, without having a major impact on their lives Johansson et al., 2015.

Theoretically the results of our study can be discussed connected to a model of reconciliation from a caring perspective Kato et al., 2016, viewing reconciliation with illness as a phenomenon that can be seen as a process. A process and a movement that will never end. Through a new whole, human beings find meaning in life and can exploit their full potential. It is crucial for registered nurses and other health-care professionals to have knowledge about the reconciliation process to be able to support patients in their journey in life with their diabetes. The theory base of our study is also in line with Forchuk et al; Gustafsson, 2008 that has explained reconciliation as a reconstruction of self or a return to an earlier way of living. They demonstrate a process that start with improvement in their inner world of thoughts and feelings and result in reconciliation with the outer world. Reaching that point of reconciliation, patients will be able to move from focus on inner self towards thinking of a larger context they previously belonged to.

There are few studies upon the process of falling ill, as well as few studies on the process of living with insulin treated diabetes from the diagnosis, present time, and future perspectives. This raises questions about how persons experience a life with insulin treated diabetes and their ability to be reconciled with the changes in life that diabetes cause.

The aim of the study was to describe experiences of the reconciliation process living with insulin treated diabetes.

## Materials and methods

Our study has a qualitative descriptive design with an inductive approach, based upon in- depth interviews with persons diagnosed with insulin treated diabetes. Qualitative content analysis, according to Graneheim and Lundman; Forchuk et al., 2003; Granheim & Lundman, 2004 was used for analysing the transcribed data. This was also guided by instructions in accordance with COREQ Graneheim et al., 2017.

### Study context and recruitment

Persons who were diagnosed with insulin treated diabetes for at least one year, were recruited from two regions, invited by “their” diabetes nurses in outpatient primary care. The diabetes nurses chose the patient with diabetes randomly which gave a rich variety in age, gender, and experiences.

### Sample

Nineteen participants, nine women and ten men, aged between 20 to 75 with a median age of 45, who had been diagnosed with insulin treated diabetes between one year and 42 years before, with a median duration of diabetes at13 years, accepted inclusion in the study (See, Table 1).

**Table 1.** Participants shown by numbers as identification in the result text.

Nr	P:1	P:2	P:3	P:4	P:5	P:6	P:7	P:8	P:9	P:10	P:11	P:12	P:13	P:14	P:15	P:16	P:17	P:18	P:19
Gender	F	F	F	F	F	M	F	M	F	M	M	M	M	M	M	M	F	F	M
Age	30	53	29	45	45	30	30	20	24	55	30	30	75	50	50	33	63	60	60
Diab duration (DD)	3	13	10	20	42	26	1	3	2	3	1	3	15	35	38	18	20	5	36

The interviews were performed and audiotaped by a former nurse included in the research team in their homes or at the diabetes clinic, depending on the participants’ choices. They were asked to describe experiences of living with diabetes. Examples of questions asked were: Can you tell me your story about living with diabetes? What does it mean? And how does it affect you? If the interviewees were uneased during the interview, they were offered follow-up by their usual caregiver in close connection to the interview.

### Data analysis

The main concepts of Graneheim and Lundmans (Forchuk et al., 2003; Granheim & Lundman, 2004) description of content analysis is naïve reading, domains, meaning units, condensations, codes, subcategories, main categories in the following process: The text was read multiple times to get an overall picture of the text through naive reading. Natural domains of the process of reconciliation from past, present, and future were shown inductively during a naive reading. This analysis ended in a unifying theme that can be described as a “red thread” running through several categories that brings meaning to the experiences various manifestations.

The researchers in the team, all RNs with many years’ experiences of clinical nursing in different settings (outpatient care—inpatient care), worked close together during the steps of the analysis. The text was divided into meaning units that responded to the purpose of the study (426 meaning units). The meaning units were chosen in a triangulation process i.e., The researchers were reading the text separately and then discussing the text together to reach consensus and secure that all topics raised at the interviews were covered. In the next step, the text was abstracted with a low degree of interpretation, to obtain the meaning of the text as a condensed meaning. The different codes were compared to similarities and differences so that no visible overlaps were found in respective codes. In the last step, the codes were brought together in subcategories whose contents could be returned to the contents of the conjugated entities. Each subcategory was named by content-characteristic words. Subcategories with similar meaning were grouped together to main categories (Forchuk et al., 2003; Granheim & Lundman, 2004). This analysis involves a back-and-forth movement between the whole and parts of the text during the whole analysis process.

### Ethical considerations

The study follows the ethical recommendations and Codex of the Swedish Research Council (Swedish Research Council, 2017) and national as well as local rules to protect participant rights, in line with the Declaration of Helsinki (Saha et al., 2013). The participants were informed with both an oral and a written consent that participation was voluntary, and that they had the right to withdraw and that all collected data would be handled confidentially. The research protocol was approved by the regional ethical committee at Örebro University hospital ref 129/03.

## Results

Our results below show the participants’ descriptions of their experiences of the reconciliation process during different time periods that appeared as domains in the interviews and are verified with quotes from the interviews (See, Table 2). The analysis finally unifying theme running through several categories whas:

**Table 2.** Summary of the main theme, the domains, categories and sub-categories of experiences of the reconciliation process during different time periods.

The main theme		
Losing your footing for a while before adapting and getting hope again		
The time domain	Category	Sub-category
At diagnosis	Striving for control	Getting insights and knowledge
		Changing life circumstances
		In need of support
In Presence	Struggling for balance	Developing strategies
		Evaluating relations to others
For Future	Avoiding or adapting	Living in the present
		Feeling threatened and Uncertain
		Taking risks
		Hoping for the best

### Losing your footing for a while before adapting and getting hope again

Time at diagnosis was a time of brooding over what the disease meant for their body and in everyday life. They felt they could develop insight into their own ill health and regain control over life with help of knowledge. They could use help from others if the help was on their own terms and based on their own individual needs. Present time was a struggle for finding balance in their body, as a person and in everyday life. Their planning strategies for their body’s needs could change their experience of themselves as a person and everyday life. Living in the present, hope for a life without diabetes complications and taking care of what one can do were described as strategies for dealing with future uncertainty.

Here we will present the process according to the domains that occurred in the text divided into main categories and its subcategories:

## At diagnosis

The first domain that appeared in the text was the time at diagnosis.

### Striving for control

The participants described internal control as a way of getting a grasp on the body. This meant experiencing any possible illness or in some cases even the recognition of diabetes.

Losing control was experienced as scary and sometimes even life-threatening. Understanding through knowledge gave a picture about what the disease could mean and gave a certain sense of control. Living with diabetes required life circumstances changes and was a balancing act between controlling and being controlled by the plasma glucose level. The participants described external control as both demands and support from others.

#### Getting insights and knowledge

Insight into ill health meant in the beginning a change in the body. This consisted of increased thirst, mood swings and fatigue. Sometimes the body was experienced as heavy. These changes to the body led to an insight into possible illness. When the diagnosis was set some saw the diagnosis as a punishment for living too good a life. They saw the diagnosis and life as a diabetic as a life with limitations. There was no value left in this life, and it felt unfair not being able to be healthy again.

Yes, first I became angry, I always am when I find out something because it felt like hell, you can’t get well. It felt like a handicap anyway. And I knew that I would have this disease my whole life (P2, F, 53, DD13)* *=Patient no 2, Female, Age 53, Diabetes duration 13 years.

Others felt that the diagnosis was something serious but was not life changing some of the participants had no insight into their illness at all they had no insight about the disease, concern, or sorrow about the situation they were in.

At first, I did not understand I had it … and we said it might not be right. But then I did not really understand what it actually meant … yes, I said OK but what do we do about his then, I thought, because I just want to go back to my job (P11, M, 30, DD1).

Inexperience concerning the procedures controlling plasma glucose level was

experienced as scary. Not having control over this could be experienced as a threat to life. Life directly after the diagnosis was experienced by many as organized by time—when should insulin be taken, or food eaten and what activities should/could be done and when? Even leaving hospital or clinic could be experienced as a threat without being prepared for a life with the disease. The participant described it as:

I did not want to go so far at first really like going home so far without staying home to see how it works, it takes some time to dare to rely on this too (P1, F, 30, DD3).

The experience of acceptance developed with time when they adapted, and the control of the plasma glucose level became a practice. A participant described it as:

Then I stepped into myself and just thought that but ok, I’ll survive this, so I must do it. That’s just the way it is (P7, F, 30, DD1).

For many, control of plasma glucose gave increased freedom and they experienced health. However, daily routines needed to be maintained as regularly as possible to balance the plasma glucose level.

The importance of knowledge became clear for them. An inaccurate or only partially accurate picture of the disease and consequences of the disease on life led to grief and anxiety in participants.

… and then I read an old medical book, and then it said that half of all babies died at birth, I felt that God, this is big, then I became very sad and went home to mom and cried … Then they soon explained that it is nothing like that (P3, F, 29, DD10).

Correcting the image and consequences of the disease on life could calm them enough to develop or gain knowledge through tailored information that could reduce fear. They felt that they could develop insight into their own ill health and regain control over life with the help of knowledge.

#### Changing life circumstances

The need for life circumstances changes was experienced as a requirement by all. Some experienced the change from before as overwhelming and life changing. They felt the change as something that changed them into something they were not. Some of the participants felt obliged to adjust their choice of occupations or working hours in line with the requirements of the disease. They discounted certain occupations because there associated risk.

I should have been a carpenter, but I jumped off for the disease … I felt so unstable att the building so I thought it might be dangerous … hard as well, now I must plan something new … now I do not know (P8, M, 20, DD3).

Some participants became insecure as to what the body could really do and felt forced to give up sports because it became too difficult to make it work in relation to the disease. Life change needs were different from participant to participant and depended on previous habits, life choices and plasma glucose level stability. Some even felt that the lifestyle change was a good thing with better and healthier habits than before the diagnosis. Other felt that it was the attitude of the individual himself that was decisive for whether it would be good to live with the disease or not.

#### In need of support

The participants described external control as both demands and support from others.

Participants felt demands on them to follow the plan for how to behave and perform as persons with diabetes. For them, support from healthcare personnel could provide security. The diabetes nurse helped to improve self-esteem by giving confirmation and permission in the decisions that needed to be made. Some participants experienced difficulties in being treated differently and being objectivized as a diagnosis in contact with healthcare professionals. They felt stigmatized, different, and sometimes more like a diagnosis than a person. Healthcare professionals expected them to be capable of understanding all tailored information about the disease and treatment and then perform the necessary change of life-circumstances on their own.

And then you get a lot of information, and you should just take everything in, and I forget a lot of it. Now this has happened in my life and they’re just sending me home and I’m supposed to fix it all by myself. All alone (P1, F, 30, DD3).

Many of the participants experienced the hospital environment as a scary environment. In addition, the hospital environment was sometimes experienced as a safe environment with competent personal who had control over the situation. They felt that support from family, friends and others in the same situation was very important.

I shared a room with a guy, and we were the same age, there was only a year between us. And he was there for the same thing I was and then we could, then it was easier to accept it in a way. Somehow, I was no longer alone (P15, M, 50, DD18).

In contrast, family and friend´s expectations based on their own knowledge could lead to them experiencing a lack of support. The inability or willingness of relatives and partners to share and support was very important in the situation, as well as the experience of stigmatization.

But at home maybe it was a bit more so that we ate food that was suitable for everyone … But if you were invited for dinner there was always an extra bowl, it was mine then … And it was often felt like constrained to eat it even if I did not think it was good. But the someone had made it special for me (P14, M, 50, DD35).

They felt that support from family and friends was very important though the willingness of others to help instead could lead to an experience of stigmatization as they needed help from others on their own terms. The person’s own willingness to open up, and talk about how they experienced the situation, how their life changed and if they were willing to receive support was also important.

## In presence

The second domain that appeared in the text was present time.

### Struggling for balance

Present time was a time when participants expressed to some extent that planning strategies were necessary and a tool to regain control in self and life. Some could use through adaptation flexible strategies for managing the consequences of the disease. It was a balance act between what the disease demands and what was perceived as acceptable for self and life. To be identified as self and not as a diabetic was important for their experiences of having regained control. Relation to others could be seen both as a burden and as a resource in life.

#### Developing strategies

The need for planning strategies was necessary for the participants. They described long-term planning strategies as tools for regaining control in self and life. Planning strategies could be stressful and controlling for some participants. For others, they were a way to a healthier and better life. Finding balance was also seen as a tool to regain control in self and life. The disease was constantly present and required long-term planning which meant controlling time for insulin and food intake choices.

What matters with this is to never be free. All days must follow a certain pattern. If something else is to be done then everything must be planned very carefully, for example. When I go out and practice for an hour a couple of times a week, I must think very carefully so that nothing goes wrong (P10, M, 55, DD3).

For others these strategies were something natural and something they had always done. Some participants saw the planning strategies as a new beginning of a healthier and much more organized life. While others need to manage the consequences of the disease.

They took away one of my legs … It was here the changes begun in with how I perceived the differences in life before and after diabetes. Because I’m one of those, or were, such an outdoor person … I will not be that anymore. So, I have tried in different ways and found out that I can replace the old possibilities to come back to my interests (P13, M, 75, DD15).

The disease required flexibility and ability from the participants to solve problems in order to continuously re-evaluate and develop strategies for managing the consequences of the disease. Many diabetics learned to recognize shift in plasma glucose levels which was perceived as a security. In some cases, diabetics couldn´t recognize the shifts to a too low plasma glucose level which affect both themselves and others with consequences that could be both scary and life threatening.

… I had to take, go over to these meal doses so I had a couple of real bangs then that I … got cramped, it was like epilepsy … I woke up at the hospital, with two policemen looking at me … (P19, M, 60, DD36).

Struggling for balance was also a struggle between the patient role and self. Being identified as self and not as a person with diabetes facilitated making life circumstances changes that were acceptable based on their own personal needs.

But I’ve told my diabetes doctor that I’m coming and doing this as I come to meet this disease halfway and then the disease will meet me halfway. And then I will do what I can and then I will think I cannot do more (P7, F, 30, DD1).

Sometimes the still ongoing changes of life required to balance the disease requirements was too difficult to do for some of the informants. Others found balance in present, integrated the disease, made the necessary changes and experienced it as if they did not even have diabetes.

#### Evaluating relations to others

Relation to others could be seen both as a burden and as a resource in life. The participants could sometimes experience it has to be a burden for others because their life circumstances changed. A burden could refer to their relative’s because they felt sorry for them. They found it hard to have a closer relationship to people who listen but not really understand what it means to live with diabetes. Ignorance from other people could lead to uncomfortable situations where the compliance with what the disease required was questioned. This led to the conclusion that the opportunity to share had limitations. Some people around them had a desire to understand and ask questions while others had silent unspoken opinions of how to live the life with diabetes:

But I think if I took a piece of cake there, I do not think anyone would say anything, but I think they would look and think (P5, F, 45, DD42).

The participants though that it was limiting mastering the disease themselves without or with insufficient support from others. They felt anxiety and vulnerable in low plasma glucose situations if no one could provide support and help them. Not having the opportunity to meet a doctor or diabetes nurse as planned or having to change appointments time after time led to insecurity. To meet others in the same situation gave new perspectives and increased security even if everyone’s situation and history of illness are individual and different.

## For future

The third domain that appeared in the text was for future.

### Avoiding or adapting

To live with diabetes as a part of life required living in the present. All participants expressed to some extent that living in the present, avoiding some thoughts about the future, was a way to live with illness as a part of life. By living in the present and keep away from some situation they could avoid speculations of a future life with complications. A life with diabetes could be acceptable if no serious complications arose which was a way of adapting to a life with diabetes. Adaptation was a way of changing or lowering previous life goals and activities in life to what was possible regarding a life with diabetes. Here the thoughts about the future can be seen as a way of viewing the way, getting glints of a reconstruction of self or a return to see themselves in earlier way of living. Or not at all seen the future so clearly if they still have a long way to go in the reconciliation process.

#### Living in the present

Through living in the present, the participants could hide and avoid speculation about the future that might still not come. To be able to live in the present they needed to avoid some situations and people who would otherwise make them aware of the possible life ahead of them. Hiding was a way of avoiding thinking about the future. Avoiding acquiring knowledge about diabetic complications could be a way of not thinking about future complications that might still not come. Avoiding could also be a way of hiding from thoughts of future complications. One informant expressed it like this:

Because I could not, I could not read about the complications. So, I’m not in the diabetes association, I do not get it, I’m shielded a little bit (P5, F, 45, DD42).

#### Feeling threatened and uncertain

A threat to health and life was something the disease induced as consequences of the disease such as impaired vision, blindness, and other complications.

When it comes there is a small sting in the heart. And there may be fear of the future, anxiety for the future and what will happen. If you are going to have complications or at worst, have a stroke. It’s the worst nightmare I have, related to diabetes, or dialysis (P9, F, 24, DD2).

The participants felt that having the future risk of getting very sick and feeling bad hanging over them was hard to get used to. Even relatives showed an unwillingness to know all the details because of fear of what might happen later. As a result, they did not provide sufficient support. Meeting other diabetic patients with consequential diseases could be scary and lead to thoughts about length of life. One way of keeping away from thinking was to try to avoid thinking about complications or avoiding situations and people who could consciously bring about these thoughts.

Uncertainty was experienced as a part of life with diabetes and strategies to keep healthy did not always help because the disease could lead to serious complications anyway. The treatment did not always make the participants feel good or encourage them to live by the rules since there was still a fear of losing bodily functions in the present.

Well, that’s the first thing you can hear and as you know, and you know that in the past, yes, or yes, even now, but many became blind and … at least get reduced vision and things like that, so you know a lot well as well and that may happen but.(P3, F, 29, DD10).

Living in the present, taking advantage of the moment and taking care of what one can do were described by participants as strategies for dealing with this uncertainty.

#### Taking risks

To take a risk was sometimes worth it and sometimes not. Higher plasma glucose level is not good for the body in the long term but may be good in the short term to facilitate life. When the plasma glucose level was too high from a medical perspective, thoughts came up about what the body was capable of and what sequelae this could lead to.

So, then that’s the question and when does it come to me? Because at the same time I know that I’ve always been a little bit higher, which is not good for the body. But it has been good for my diabetes and me (P15, M, 50, DD38).

Being spontaneous by eating without planning could lead to experiences of guilt. For some, the thought of complications could lead to a sense of despair about the situation.

But if I´m going to like that and then, do not take a little piece of cake or something, and I´m not prepared, and do it then I get bad conscience. And the I think no, I would not have done, now I ruined a bit of thi, now, I´ll die sooner and then I feel bad (P18, M, 60, DD36).

Sometimes the adaptation to what the disease required refrained them from certain things in life, in other cases was the risk of future complications worth taking. They could accept a life with diabetes if no serious consequential illnesses arose which was a way to adapt to a life with diabetes.

#### Hoping for the best

To hope was to hope for a future without serious complications or to get rid of the disease. They hoped that they would be able to share both new research findings to simplify everyday life, as well as research and treatment methods that prevent complications of the disease before any complications began to evolve. Most of all, they hoped to get rid of the disease by finding solutions to the cause of the disease or being able to undergo a transplant. A participant describes it as:

Still, my biggest wish is to get a new pancreas. It is always on my mind that I want to get this out of my body (P2, F, 53, DD13).

If this wasn’t possible, they hoped in any case that the body would continue to function and that they did not have serious complications to their diabetes.

## Discussion

Understanding the reconciliation process will enable nurses to plan and provide professional care (Gustafsson, 2008; Kato et al., 2016) tailored to promote flexible regimes that facilitate quality of life for patients living with diabetes. The experience of the phenomena as losing their footing for a while before adapting and getting hope again were a unifying theme running through the informants’ descriptions. The result of our study is divided into three domains since the text showed that the experiences differed related to time. The time at diagnosis seemed to be a turbulent one, due to discovering that you have a longstanding and incurable disease. In the domain in presence the results show that diabetes requires development of flexible strategies for managing the consequences the disease has on self and life. While viewing the future, the seemingly extremes avoidance or adapting became the focus. The result of our study shows that the participants losing their footing for a while before adapting and getting hope again.

At time for diagnosis, we found that the participants felt that something was wrong in their body and not in any way natural which differs from another Swedish study in which diabetes was explained by natural causes (Johansson et al., 2009). Our study showed, however, that family members and friends with diabetes could contribute to an understanding of their illness and that they therefore could assimilate their diagnoses, which is consistent with Johansson et.al. 5 In the domain focusing present time the topic was about struggling for balance, developing strategies for that but also evaluating their relationship to others.

Our results show that the emotional crisis which some of the persons with diabetes were at the time of the diagnosis made it difficult for them to collect or manage the information provided to them. Tailored information could help people to develop a flexible regime 9,10. This is also supported by the results of our study, provided that the person with diabetes is susceptible to information. Tailored information was especially important for persons with diabetes at diagnosis, because of the lack of insights and knowledge many experienced.

Persons at earlier stages of change need to find positive reasons for change. Once this stage is completed persons must believe in the ability to change to increase self-efficacy (Mishalia et al., 2011). The results from our study show that persons with diabetes need tailored and long-term support from healthcare professionals and especially from the diabetes nurse. Initially, in the hospital, several experienced that they had good support from co-workers, especially other persons with diabetes of their own age and with similar experiences that they could reflect on. When they received that kind of support, based on their own needs, they felt that they were not alone in their situation. Previous studies reflect the results in our study, showing that both short- and long-term support from both professionals and relatives are important for the patient’s self-management ability and diabetes empowerment (Isaksson et al., 2015). Support from friends of the same age is important (Huston et al., 2016; Scholes et al., 2012) and together with illness experience, personal beliefs and life situation, social support is described as important for the integration of self-management (Audulv et al., 2012)

Our study’s results show that the diagnosis became clear when persons with diabetes began to be treated differently, more as a diagnosis than as a person by healthcare personnel. Even people around them sometimes stigmatized the participants instead of supporting them. The essential meaning of falling ill with diabetes can be a fight for not becoming one’s illness (Johansson et al., 2009). The participants in our study described that they started fighting in the hospital and then continued the fight in private life. As soon as they were diagnosed, they were supposed to understand, learn, and adapt to the new situation with diabetes even if they did not experience that they had the prerequisites for doing so. Adaptation is a way of going on with life and feeling well. Adaptation can be described as false reconciliation when persons with diabetes have no other choice than to adapt to their illness (Johansson et al., 2009). The results from our study show that when persons with diabetes developed insight into their own health and had knowledge about how to handle the situation, normal daily routines became easier to handle. Some of them even saw benefits because their habits were better than before the diagnosis. Degrees of acceptance as a step of reconciliation were connected to whether they could look upon their life beyond the disease or not. Some explain that their own attitude was vital in determining their ability to accept a life with diabetes. Our results show though, that some of the participants couldn’t accept their situation, they could only manage it. This led them to not being open to life or being connected to others on a deeper level. Handling necessary lifestyle changes was harder for participants who were not able to be united with others for support.

Our study shows that knowledge useful for understanding the illness may be a prerequisite for reconciliation for persons with diabetes. Johansson et al. (Johansson et al., 2016) show in their study that for patients with newly diagnosed diabetes, the stories of others could be an initiator in the search for knowledge. Through reconciliation the person can leave the old behind and become a new transformed unit (Mishalia et al., 2011)

According to Johansson et al. (Mishali et al., 2011) living with diabetes means taking responsibility for balancing glucose levels and incorporating the illness into life with necessary knowledge and sensitivity to changes. Our results, in the domain at present, show that it was necessary to develop long-term planning strategies struggling for balance and that it took time to do so. For some it could be problematic due to fast-changing and unstable plasma glucose levels. For others it could be stressful and controlling because they needed to make such big changes in their life and lifestyle. Persons with diabetes need both individual information and support on their own terms to take control over self and life (Mishali et al., 2011). Our results show that individual information and support is not enough. There was a need to develop an individual and flexible approach as well as the importance of continuously re-evaluating and developing strategies for managing the consequences of the disease. That need leads to a willingness to learn, both alone and with others (Mishali et al., 2011). Living with diabetes can be seen as an ongoing process to address the consequences of the disease for self and life. Our results are consistent with (Mishalia et al., 2011) which looks upon reconciliation as a process that can never be ended or fulfilled, although an individual approach gives persons with diabetes tools for managing the consequences of the disease.

The participants described their relation to self as being happy for the opportunities for a good life and hoping for limited losses. Being identified as self and developing a positive attitude to life was important for the informants’ experiences of regaining control. People’s attitudes can affect their ability to accept diabetes and live with it as a part of themselves (Johansson et al., 2015). Based on their reasoning, a more positive attitude to the illness could create conditions for acceptance and maybe even reconciliation, especially if their life became better and healthier than it was before diabetes.

When diabetes is integrated, the person is changed, and the illness is involved in the body. Life thus forms a new way of being (Kato et al., 2016; Kneck et al., 2012). Becoming means that through reconciliation, persons can overcome their fear and through the change in self and life, create new conditions for relationships with others (Kato et al., 2016). Reconciliation is a prerequisite for being able to find balance and harmony in life as well as to become united with others (Gustafsson, 2008). Our study shows that patients with diabetes had varying relationships with others. Some relationships gave new perspectives and support while others treated them differently or blamed them for the situation. However, many thought that no one without diabetes could really understand what living with diabetes really meant which made contact with others on a deeper level difficult. The results of our study show that there were shortcomings in the reconciliation process and that the balance and harmony of suffering was not fully achieved for many persons with diabetes. Flexible and suitable strategies were not even always enough to control the consequences of the disease.

Treatment is necessary for a good life living with diabetes. It takes time to understand the need for planning strategies which can often be experienced as a deterioration of autonomy. Despite this, it is necessary to take early control of the disease to avoid damaging the body later in life (Johansson et al., 2009). Our study’s results show that planning strategies were a way of regaining control. It was also important not to become your illness. Our results therefore show that there is a balancing act between planning and autonomy. Taking control and responsibility for illness can lead to higher levels of autonomy, which indicates the importance of taking control and responsibility for not becoming your illness. This higher level of autonomy could lead to a more supportive role from the healthcare professional and better self-management from people with diabetes (Tan P et al., 2012).

The participants in our study needed to develop a flexible regime to facilitate both the quality of life and medical outcomes as described by Frost et al. (Johansson et al., 2009) The result of our study shows that quality of life or some aspects of it were sometimes even more important than low plasma glucose level from a medical perspective and a risk worth taking regarding the risk for future complications. Quality of life was also something that happened in the present, while they knew nothing about the **future**. Instead, they put their hopes on science and hoped it could provide other possibilities and treatment options later in life. Persons with diabetes need developing preventing strategies to avoid hypoglycaemic events in the short term (Tan P et al., 2012). Our study, however, shows that some of the participants dared not take the risk and tried to live a life that followed treatment advice even though it affected their quality of life in a negative way. Whether they took risks or followed the ordinances they had received, only the future will tell if they suffer from complications or not and how much uncertainty they must try to live with. This kind of uncertainly could be an obstacle to be reconciled when fear of future complications is always there.

Even if the study illustrates the problems for persons with diabetes in achieving a more developed and stable inner form of reconciliation, as described by, (Mishalia et al., 2011) they continuously struggled in the reconciliation process. This healing process worked in a linear way between past, present, and future. Even if the participants found it hard to completely reconcile with a life with diabetes, they developed their knowledge and competence using flexible strategies. This is what Gustafsson (Kato et al., 2016) calls an outer form of reconciliation. Life with diabetes was experienced as largely an acceptable life in the present time. This is what Gustafsson (Kato et al., 2016) calls an outer form of reconciliation. Life with diabetes was experienced as largely an acceptable life in the present time. Understanding the reconciliation process will enable nurses to plan and provide professional care (Gustafsson, 2008; Kato et al., 2016) tailored to promote flexible regimes that facilitate quality of life for patients living with diabetes.

## Methodological considerations

In our study the sample size was rather large for being a qualitative in-depth interview study but still, according to Graneheim and Lundmans description (Forchuk et al., 2003) suitable for finding variations in descriptions as well as common meaning of the phenomena of interest. To increase credibility, and variation of data the included patients were of mixed age and sex. They had been diagnosed with diabetes mellitus for between 1–42 years before the data collection. All participants in the study received insulin treatment from the time of diagnosis. In qualitative research, authors’ preunderstandings may affect the interpretations (Tufford & Newman, 2012). The authors were careful to ensure that they maintained control over their preunderstanding to avoid a biased comprehension as much as possible. Though, the analytic process and the different views in the researcher team could influence the analyses. Maybe it is impossible not to be biased due to former experience, but to have a mix of individuals with different experiences of nursing practice will at least partly counteract bias. Graneheim et al. (Granheim & Lundman, 2004) discuss the relationship between interpretation and abstraction and that both the purpose of the study and the quality of data are crucial in determining the level of interpretation and abstraction that can be performed. Based on their two-dimensional model of abstraction levels and interpretation degrees, our study has a low abstraction level. During the analysis, our approach was close to descriptions of experiences and on a concrete analytical level. Regarding transferability, the findings may be transferred to other contexts (Gustafsson, 2008; Kato et al., 2016), though each reconciliation process is deeply personal.

## Conclusions

When persons with diabetes developed insight into their own health, and had the knowledge to handle the situation, daily life routines became easier to handle. The degrees of acceptance were connected to whether they could look upon their life beyond the disease or not. There was a need to develop a flexible regime that facilitates both quality of life and medical outcomes to reach reconciliation. In the outer form of reconciliation, were shown as a transformation of themselves and their strategies continuously over time. In the inner form of reconciliation, the threat of future complications constitutes an obstacle to reconciliation.
